# Forecasting with a Bivariate Hysteretic Time Series Model Incorporating Asymmetric Volatility and Dynamic Correlations

**DOI:** 10.3390/e27070771

**Published:** 2025-07-21

**Authors:** Hong Thi Than

**Affiliations:** Faculty of Mathematics and Statistics, Ton Duc Thang University, Ho Chi Minh City 700000, Vietnam; thanthihong@tdtu.edu.vn

**Keywords:** bivariate Student’s t-distribution, hysteresis, asymmetry structures in volatility, Markov chain Monte Carlo, value-at-risk, marginal expected shortfall, out-of-sample forecasting

## Abstract

This study explores asymmetric volatility structures within multivariate hysteretic autoregressive (MHAR) models that incorporate conditional correlations, aiming to flexibly capture the dynamic behavior of global financial assets. The proposed framework integrates regime switching and time-varying delays governed by a hysteresis variable, enabling the model to account for both asymmetric volatility and evolving correlation patterns over time. We adopt a fully Bayesian inference approach using adaptive Markov chain Monte Carlo (MCMC) techniques, allowing for the joint estimation of model parameters, Value-at-Risk (VaR), and Marginal Expected Shortfall (MES). The accuracy of VaR forecasts is assessed through two standard backtesting procedures. Our empirical analysis involves both simulated data and real-world financial datasets to evaluate the model’s effectiveness in capturing downside risk dynamics. We demonstrate the application of the proposed method on three pairs of daily log returns involving the S&P500, Bank of America (BAC), Intercontinental Exchange (ICE), and Goldman Sachs (GS), present the results obtained, and compare them against the original model framework.

## 1. Introduction

Shocks to a time series can cause persistent effects, whereby the influence of disturbances spreads and persists over time. This phenomenon, referred to as the hysteresis effect, reflects a form of path dependence in which system dynamics respond asymmetrically to past shocks. To address issues related to excessive or spurious regime shifts, a range of univariate hysteretic time series models have been developed by the authors of [1,2,3,4,5,6,7]. In financial econometrics, it is well established that asset returns tend to exhibit co-movement. Understanding and forecasting the temporal dependence in the second-order moments of these returns is a key concern in finance. Multivariate models provide a useful framework for capturing complex features such as volatility clustering across multiple assets, time-varying correlations, and joint downside tail risks across industries. These considerations have led researchers to extend univariate volatility models into the multivariate setting. For instance, the authors of [8] introduced the VECH and BEKK models, while the authors of [9] proposed the Dynamic Conditional Correlation (DCC) model, which allows for a time-varying conditional correlation matrix. In contrast, the authors of [10] developed a model that captures correlation dynamics through a weighted average of past correlation matrices, reflecting the persistence of conditional correlations. The authors of [11] developed an asymmetric Dynamic Conditional Correlation (AG-DCC) model to examine the presence of asymmetric responses in conditional volatility and correlation between financial asset returns, particularly allowing for asymmetries in the correlations. A comprehensive discussion on generalized univariate volatility models can be found in [12]. The authors of [13] suggested an extension of [10] using a Bayesian Markov chain Monte Carlo (MCMC) technique to accommodate heavy-tailed distributions. Nonetheless, these models do not consider regime-switching behavior, which is potentially essential for modeling structural shifts and regime-dependent dynamics in financial markets.

In the multivariate context, the authors of [14] proposed the Hysteretic Vector Autoregressive (HVAR) model, which incorporates delayed regime switching based on a hysteresis variable. Specifically, transitions between regimes occur only when this variable exits a predefined hysteresis zone. The authors of [15] introduced a bivariate HAR model incorporating GARCH errors and time-varying correlations. This model integrates features of dynamic correlation, asymmetric effects on correlation and volatility, and heavy-tailed distribution within the multivariate HAR framework previously developed by [14]. However, the asymmetry in [15] is introduced only through the regime-switching behavior of a hysteresis variable within the system. This approach overlooks the leverage effects associated with individual asset returns, which have been emphasized in earlier studies in [11]. In a univariate framework, the authors of [16,17] examined the intricate dynamics between financial returns and volatility, emphasizing the asymmetric effects of shocks. The authors of [16] modified the GARCH model to account for seasonal volatility patterns, differential impacts of positive and negative return innovations, and the influence of nominal interest rates on conditional variance. Similarly, the author of [17] generalized the ARCH framework by modeling the conditional variance as a quadratic function of past innovations, allowing for a nuanced capture of volatility patterns, including asymmetries and leverage effects. Both studies underscore the importance of accommodating asymmetries in volatility modeling to better understand and predict financial market behaviors.

As a result, the volatility specification in [15] leaves room for improvement in modeling asymmetric effects at the level of individual return series. In this paper, we develop an extension of the multivariate hysteretic autoregressive (MHAR) model with GARCH errors and dynamic correlations (see [15]) to accommodate asymmetries in volatility dynamics. Specifically, we incorporate two well-known asymmetric volatility specifications: the GJR-GARCH, as defined in [16], and the QGARCH, proposed in [17]. These extensions result in two model variants, namely, the MHAR–GJR–GARCH and the MHAR–QGARCH models.

To the best of our knowledge, this is the first study to explore asymmetric volatility structures within the MHAR–GARCH framework. By introducing these asymmetric components, the proposed models offer greater flexibility in capturing the heterogeneity and nonlinear behavior commonly observed in financial asset returns. Such flexibility is particularly important in modeling risk dynamics, especially during periods of market turbulence where asymmetries in volatility play a crucial role. Based on the proposed models, we employ an adaptive multivariate t-distribution to account for heavy-tailed errors, and utilize the SMN representation (see [18]) to flexibly model marginal error distributions with varying degrees of freedom, improving the model’s fit to the target time series.

In finance, systemic risk refers to the possibility that problems in one financial institution or a group of them could spread throughout the financial system due to the strong connections between institutions. Such a chain reaction can lead to serious disruptions or even cause the entire market to collapse. Following [19], the Marginal Expected Shortfall (MES) is employed to empirically evaluate the extent to which this risk measure addresses practical concerns related to systemic risk, using a large sample of major U.S. banks. In this study, we consider two widely used risk measures: Value at Risk (VaR) and MES, in which MES plays a more prominent role in capturing tail risk and systemic vulnerability. Additionally, we implement two backtesting procedures to assess the accuracy of out-of-sample VaR forecasts.

A major limitation of the proposed models lies in their increasing complexity, particularly due to the large number of parameters that must be estimated and the challenges involved in modeling nonlinear multivariate structures. As the nonlinearity and asymmetric structures of the proposed models increase, traditional estimation methods become inefficient or impractical. To overcome these difficulties, we adopt a Bayesian framework using Markov chain Monte Carlo (MCMC) techniques, which allows for simultaneous inference of all unknown parameters.

The remainder of this paper is divided into the following sections: The multivariate hysteretic autoregressive model with time-varying correlations and asymmetry structures in volatility is presented in Section 2. Bayesian inference for the model parameters is presented in Section 3. Forecasting VaR and the marginal expected shortfall (MES) are considered in Section 4. Section 5 examines simulation. The empirical study is described in Section 6 and marks are covered in Section 7.

## 2. Multivariate Hysteretic Autoregressive Model with Asymmetry Structures in Volatility and Time-Varying Correlation

We consider the MHAR–GARCH model, a multivariate hysteretic autoregressive model with GARCH type errors:

(1) $$\begin{array}{ccc} y_{t} & = & \Phi_{0}^{\left(J_{t}\right)}+\sum_{l=1}^{c}\Phi_{l}^{\left(J_{t}\right)}y_{t-l}+a_{t},\\ a_{t} & = & \mathrm{diag}\left(\sqrt{h_{1,t}},\ldots ,\sqrt{h_{k,t}}\right)\epsilon_{t}\;\mathrm{where}\;\;\epsilon_{t}∼D\left(0,\Sigma_{t}\right),\\ h_{i,t} & = & \omega_{i0}^{\left(J_{t}\right)}+\sum_{l=1}^{p_{i}}\alpha_{il}^{\left(J_{t}\right)}a_{i,t-l}^{2}+\sum_{l=1}^{q_{i}}\beta_{il}^{\left(J_{t}\right)}h_{i,t-l},\;\;\;i=1,\ldots ,k,\\ \Sigma_{t} & = & \left(1-\theta_{1}^{\left(J_{t}\right)}-\theta_{2}^{\left(J_{t}\right)}\right)\Sigma^{\left(J_{t}\right)}+\theta_{1}^{\left(J_{t}\right)}\Sigma_{t-1}+\theta_{2}^{\left(J_{t}\right)}\Psi_{t-1},\\ J_{t} & = & \left\{\begin{array}{cc} 1 & \mathrm{if}z_{t}\leq r_{L}\\ 2 & \mathrm{if}z_{t}>r_{U}\\ J_{t-1} & \mathrm{otherwise},\;\;r_{L}<r_{U}, \end{array}\right. \end{array}$$

and the (u,v)th element of $\Psi_{t-1}$ is formulated as:

(2) $$\psi_{uv,t-1}=\frac{\sum_{s=1}^{S}\epsilon_{u,t-s}\epsilon_{v,t-s}}{\sqrt{\left(\sum_{s=1}^{S}\epsilon_{u,t-s}^{2}\right)\left(\sum_{s=1}^{S}\epsilon_{v,t-s}^{2}\right)}},\;1\leq u<v\leq S^{★}\leq S.$$

where $y_{t}={\left(y_{1,t},\ldots ,y_{k,t}\right)}^{\prime }$ is a vector of k assets at time t, $J_{t}$ is a regime indicator, $\Phi_{0}^{\left(J_{t}\right)}$ is a *k*-dimensional vector, $\Phi_{l}^{\left(J_{t}\right)}$ is a k×k matrix, $\Sigma^{\left(J_{t}\right)}$ is a k×k positive-definite matrix with diagonal elements, scalar parameters are satisfied $\theta_{1}^{\left(J_{t}\right)},\theta_{2}^{\left(J_{t}\right)}>0$ and $0<\theta_{1}^{\left(J_{t}\right)}+\theta_{2}^{\left(J_{t}\right)}<1$, and $\Psi_{t-1}$ is a k×k sample correlation matrix of shocks from t−S,…,t−1 for a pre-specified *S*. Moreover, $z_{t}$ is a hysteresis variable. In this study, we investigate two distinct forms of asymmetric volatility within the framework of a multivariate hysteretic autoregressive (MHAR) model. The first approach incorporates the asymmetric volatility structure proposed by [16] into the MHAR–GARCH framework, resulting in the MHAR–GJR–GARCH model. The second approach introduces the quadratic GARCH specification, as developed by the authors of [17], leading to the formulation of the MHAR–QGARCH model. We also derive the volatility dynamics of the MHAR–GJR–GARCH model:

$$\begin{array}{ccc} h_{i,t} & = & \omega_{i0}^{\left(J_{t}\right)}+\sum_{l=1}^{p_{i}}\alpha_{il}^{\left(J_{t}\right)}a_{i,t-l}^{2}+\sum_{l=1}^{q_{i}}\beta_{il}^{\left(J_{t}\right)}h_{i,t-l}+\sum_{l=1}^{r_{i}}\delta_{il}^{\left(J_{t}\right)}I_{i,t-l}\left\{\epsilon_{t-l}<0\right\}a_{i,t-l}^{2},\;i=1,\dot{...},k, \end{array}$$

where $I_{i}\left(.\right)$ is a k×1 indicator function that returns a value of 1 when the argument is true or 0 otherwise. The volatility of the MHAR - QGARCH model is as follows:

$$\begin{array}{ccc} h_{i,t} & = & \omega_{i0}^{\left(J_{t}\right)}+\sum_{l=1}^{p_{i}}\alpha_{il}^{\left(J_{t}\right)}a_{i,t-l}^{2}+\sum_{l=1}^{q_{i}}\beta_{il}^{\left(J_{t}\right)}h_{i,t-l}+\sum_{l=1}^{r_{i}}\delta_{il}^{\left(J_{t}\right)}a_{i,t-l},\;i=1,\dot{...},k, \end{array}$$

We now consider the basic cases of two models: the bivariate HAR(1)–GJR–GARCH(1,1) model and the bivariate HAR(1)–QGARCH(1,1) model. We assume that innovations in Equation (1) follow the modified bivariate Student’s-t distribution (see [15]). In this case, we apply the scale SMN representation (see [18]) to the adapted bivariate Student’s-t distribution, $T_{2}^{*}\left(0,\Sigma_{t},\nu \right)$, and we choose $z_{t}=y_{1,t-d}$. Then, the bivariate HAR(1)–GJR–GARCH(1,1) model is described as follows:

(3) $$\begin{array}{ccc} y_{t} & = & \Phi_{0}^{\left(J_{t}\right)}+\Phi_{1}^{\left(J_{t}\right)}y_{t-1}+a_{t},\\ a_{t} & = & \mathrm{diag}\left(\sqrt{h_{1,t}},\sqrt{h_{2,t}}\right)\epsilon_{t},\\ \epsilon_{t}|\Lambda_{t} & ∼ & N_{2}\left(0,\Lambda_{t}^{-1/2}\Sigma_{t}\Lambda_{t}^{-1/2}\right),\\ \Lambda_{t}^{1/2} & = & \mathrm{diag}\left(\sqrt{\lambda_{1,t}},\sqrt{\lambda_{2,t}}\right),\;\lambda_{i,t}∼Ga\left(\frac{\nu_{i}}{2},\frac{\nu_{i}}{2}\right),\;i=1,2,\\ \Sigma_{t} & = & \left(1-\theta_{1}^{\left(J_{t}\right)}-\theta_{2}^{\left(J_{t}\right)}\right)\Sigma^{\left(J_{t}\right)}+\theta_{1}^{\left(J_{t}\right)}\Sigma_{t-1}+\theta_{2}^{\left(J_{t}\right)}\Psi_{t-1},\\ J_{t} & = & \left\{\begin{array}{cc} 1 & \mathrm{if}y_{1,t-d}\leq r_{L},\\ 2 & \mathrm{if}y_{1,t-d}>r_{U},\\ J_{t-1} & \mathrm{otherwise}, \end{array}\right. \end{array}$$

where the (u,v)th element of $\Psi_{t-1}$ is described in (2) and the conditional volitilities as follows:

$$\begin{array}{ccc} h_{i,t} & = & \omega_{i0}^{\left(J_{t}\right)}+\alpha_{i1}^{\left(J_{t}\right)}a_{i,t-1}^{2}+\beta_{i1}^{\left(J_{t}\right)}h_{i,t-1}+\delta_{i1}^{\left(J_{t}\right)}I_{i,t-1}\left\{\epsilon_{t-1}<0\right\}a_{i,t-1}^{2},\;i=1,2, \end{array}$$

where $I_{i}\left(.\right)$ is a 2×1 indicator function that returns a value of 1 when the argument is true or 0 otherwise. The positivity and stationarity conditions for volatility are given as follows:

(4) $$\begin{array}{c} \omega_{i0}^{\left(J_{t}\right)}>0,\alpha_{i1}^{\left(J_{t}\right)}\geq 0,\beta_{i1}^{\left(J_{t}\right)}\geq 0,\delta_{i1}^{\left(J_{t}\right)}\geq 0\;\mathrm{and}\;\alpha_{i1}^{\left(J_{t}\right)}+\beta_{i1}^{\left(J_{t}\right)}+\delta_{i1}^{\left(J_{t}\right)}\leq 1. \end{array}$$

The bivariate HAR(1)–QGARCH(1,1) model modifies the conditional volatilities as follows:

$$\begin{array}{ccc} h_{i,t} & = & \omega_{i0}^{\left(J_{t}\right)}+\alpha_{i1}^{\left(J_{t}\right)}a_{i,t-1}^{2}+\beta_{i1}^{\left(J_{t}\right)}h_{i,t-1}+\delta_{i1}^{\left(J_{t}\right)}a_{i,t-1},\;i=1,2, \end{array}$$

where the positivity and stationarity conditions for volatility are given as follows:

(5) $$\begin{array}{c} \omega_{i0}^{\left(J_{t}\right)}>0,\alpha_{i1}^{\left(J_{t}\right)}\geq 0,\beta_{i1}^{\left(J_{t}\right)}\geq 0,\delta_{i1}^{\left(J_{t}\right)}\geq 0,{\left(\delta_{i1}^{\left(J_{t}\right)}\right)}^{2}\leq 4\left(1-\alpha_{i1}^{\left(J_{t}\right)}-\beta_{i1}^{\left(J_{t}\right)}\right),\\ \;\mathrm{and}\;\alpha_{i1}^{\left(J_{t}\right)}+\beta_{i1}^{\left(J_{t}\right)}\leq 1, \end{array}$$

and we specify the unconditional correlation matrix $\Sigma^{\left(J_{t}\right)}$:

$$\begin{array}{ccc} \Sigma^{\left(J_{t}\right)} & = & \left[\begin{array}{cc} 1 & \rho^{\left(J_{t}\right)}\\ \rho^{\left(J_{t}\right)} & 1 \end{array}\right]. \end{array}$$

## 3. Bayesian Inference

To estimate the unknown parameters of the proposed models in a Bayesian framework, for example, we create groups of the unknown parameters: (i) $\phi_{i}^{\left(J_{t}\right)}={\left(\phi_{i0}^{\left(J_{t}\right)},\phi_{i1}^{\left(J_{t}\right)},\phi_{i2}^{\left(J_{t}\right)}\right)}^{\prime },i,J_{t}=1,2$; (ii) $r={\left(r_{L},r_{U}\right)}^{\prime }$; (iii) $\nu ={\left(\nu_{1},\nu_{2}\right)}^{\prime }$; (iv) $\rho ={\left(\rho^{\left(1\right)},\rho^{\left(2\right)}\right)}^{\prime }$; (v) $\gamma_{i}^{\left(J_{t}\right)}={\left(\omega_{i0}^{\left(J_{t}\right)},\alpha_{i1}^{\left(J_{t}\right)},\beta_{i1}^{\left(J_{t}\right)},\delta_{i1}^{\left(J_{t}\right)}\right)}^{\prime }$, $i,J_{t}=1,2$; (vi) $\eta^{\left(J_{t}\right)}={\left(\theta_{1}^{\left(J_{t}\right)},\theta_{2}^{\left(J_{t}\right)}\right)}^{\prime },$ and (vii) *d*. We define θ as a vector of all the unknown parameters of the proposed model. Following that, the bivariate HAR(1)–GJR–GARCH(1,1) and bivariate HAR(1)–QGARCH(1,1) models’ conditional likelihood functions are given by:

(6) $$\begin{array}{ccc} \ln\left(L\left(y|\theta \right)\right) & \propto & \sum_{t}\{\sum_{J_{t}=1}^{2}[-0.5\;ln\left(\frac{h_{1,t}h_{2,t}\left(1-{\rho^{\left(J_{t}\right)}}^{2}\right)}{\lambda_{1,t}\lambda_{2,t}}\right)\\ \; & \; & -\frac{1}{2(1-{\rho^{\left(J_{t}\right)}}^{2})}\left(\frac{\lambda_{1t}a_{1t}^{2}}{h_{1,t}}+\frac{\lambda_{2t}a_{2t}^{2}}{h_{2,t}}-\frac{2\rho^{\left(J_{t}\right)}a_{1t}a_{2t}\sqrt{\lambda_{1,t}\lambda_{2,t}}}{\sqrt{h_{1,t}}\sqrt{h_{2,t}}}\right)]\}, \end{array}$$

where $a_{t}=y_{t}-\Phi_{0}^{\left(J_{t}\right)}-\Phi_{1}^{\left(J_{t}\right)}y_{t-1}$.

We set up prior distributions for the unknown parameters. Assume that $\phi_{i}^{\left(J_{t}\right)}∼N_{3}\left(\mu_{0i},\Sigma_{0i}^{-1}\right),$$i,J_{t}=1,2$; for the threshold parameter $r_{L}∼\mathrm{Unif}\left(l_{1},l_{2}\right)$, where $l_{1}$ and $l_{2}$ are the *p*th and (100−2p)th percentiles of the observed time series, respectively, for 0<p<33. Furthermore, $r_{U}|r_{L}∼\mathrm{Unif}\left(u_{1},u_{2}\right),$ where $u_{2}$ is the (100−p)th percentile and $u_{1}=r_{L}+c^{*}$ for $c^{*}$ is a selected number that ensures $r_{L}+c^{*}\leq r_{U}$ and at least p% of observations are in the range $\left(r_{L},r_{U}\right)$. For the degrees of freedom, we assume the scale mixture variables $\lambda_{i,t}∼Ga\left(\nu_{i}/2,\nu_{i}/2\right)$ and $\nu_{i}∼\mathrm{Unif}\left(2,60\right),\;i=1,2$, and $\rho^{\left(J_{t}\right)}∼\mathrm{Unif}\left(-1,1\right)$. For the lag *d*, we choose the discrete uniform prior $p\left(d\right)=1/d_{0}$ with maximum delay $d_{0}$. In terms of the volatility parameters, $\gamma_{i}^{\left(J_{t}\right)}$ follows a uniform distribution such that $\gamma_{i}^{\left(J_{t}\right)}$ is proportional to $I(S_{1})$ or $I(S_{2})$, where $S_{1}$ and $S_{2}$ are the sets that satisfy (4) and (5), respectively.

The conditional posterior distribution for each group is proportional to the conditional likelihood function multiplied by the prior density for that group, as shown below:

$$P\left(\theta_{l}|y,\theta_{-l}\right)\propto L\left(y|\theta \right)P\left(\theta_{l}|\theta_{-l}\right),$$

where $\theta_{l}$ is each parameter group, $P(\theta_{l})$ is its prior density, and $\theta_{-l}$ is the vector of all parameters, except $\theta_{l}$. The conditional posterior distribution of the delay lag *d* follows a multinomial distribution with a probability:

$$\begin{array}{c} \mathrm{Pr}\left(d=i|y,\theta_{-d}\right)=\frac{p\left(y|d=i,\theta_{-d}\right)\mathrm{Pr}\left(d=i\right)}{\sum_{j=1}^{d_{0}}p\left(y|d=j,\theta_{-d}\right)\mathrm{Pr}\left(d=j\right)},i=1,\ldots ,d_{0}. \end{array}$$

In this study, with the exception of the lag parameter *d*, the conditional posterior distributions of the remaining parameter groups exhibit non-standard forms. To make a statistical inference, we employ an adaptive Markov chain Monte Carlo (MCMC) method for selected parameter groups, complemented by a random walk Metropolis algorithm. Specifically, we assume that the innovation term in Equation (3) follows a Gaussian distribution, which serves as the kernel for sampling $\phi_{i}^{\left(J_{t}\right)}$. For the parameter groups $\eta^{\left(J_{t}\right)}$ and $\gamma_{i}^{\left(J_{t}\right)}$, where $i,J_{t}=1,2$, an adaptive MCMC approach is utilized to draw samples, whereas the remaining parameters are updated using the random walk Metropolis algorithm. The detailed procedures of the adaptive Metropolis–Hastings MCMC algorithm are thoroughly presented by the authors of [1,15], where the authors provide a comprehensive framework for its implementation and application. Following the guidelines of [1], we further adjust the scale matrix to attain ideal acceptance rates of 20% to 60%.

In a Bayesian framework, we need to set up the initial values for each parameter group. For the autoregressive coefficient parameters, $\phi_{i}^{\left(J_{t}\right)}=\left(0,0,0\right)$; for the degrees of freedom $\nu_{i}^{\left(J_{t}\right)}=50$; $\rho_{i}^{\left(J_{t}\right)}=0.5$; $d_{0}=3$; $\omega_{i0}^{\left(J_{t}\right)}=\alpha_{i1}^{\left(J_{t}\right)}=\beta_{i1}^{\left(J_{t}\right)}=\delta_{i1}^{\left(J_{t}\right)}=0.1$; and $\left(\theta_{1}^{\left(J_{t}\right)},\theta_{2}^{\left(J_{t}\right)}\right)=\left(0.05,0.2\right)\;\mathrm{for}\;\;i,$$J_{t}=1,2$; thresholds $r_{L}$ and $r_{U}$ are established at the 33rd and 67th percentiles, respectively; and we set p=20 to make certain of having enough observations in each regime for a valid inference. For the remainder of the analysis, we specify S=3.

## 4. Forecasting the Marginal Expected Shortfall and Value at Risk

The Value-at-Risk (VaR) and Marginal Expected Shortfall (MES) are now considered systemic risk assessments for financial risk management. The authors of [19] define MES as a financial firm’s marginal contribution to the financial system’s expected shortfall. The authors of [20] define MES at the alpha level for a financial institution at time t given $F_{t-1}$ as follows:

(7) $$\begin{array}{ccc} \; & \; & {\mathrm{MES}}_{t}=E\left[y_{2,t}\;|\;y_{1,t}<{\mathrm{VaR}}_{1,t}\left(\alpha \right);F_{t-1}\right], \end{array}$$

where ${\mathrm{VaR}}_{1,t}\left(\alpha \right)$ is the VaR of $y_{1,t}$ at the α-level such that $P\left(y_{1,t}<{\mathrm{VaR}}_{1,t}\left(\alpha \right)|F_{t-1}\right)=\alpha$. Here, $y_{2,t}$ stands for the stock return of a financial institution, whereas $y_{1,t}$ stands for the market return.

To produce ${\mathrm{MES}}_{t}$, we estimate one-step-ahead quantiles and volatilities for $y_{1,n+1}$ from the investigated model described in (3) with forecast origin t=n. We obtain quantiles from the posterior predictive distribution, which is:

(8) $$p\left(y_{n+1}|F_{n}\right)=\int_{\theta \in \Theta }p\left(y_{n+1}|F_{n},\theta \right)p\left(\theta |F_{n}\right)d\theta .$$

Suppose that $\left\{\theta^{\left[r\right]},r=n_{0}+1,\ldots ,N\right\}$ are the *r*th MCMC drawn from the posterior density $p(\theta |F_{n})$ after the $n_{0}$ burn-in sample. Thus, we can sample $\left\{y_{n+1}^{\left[r\right]},r=n_{0}+1,\ldots ,N\right\}$ from the marginal predictive distribution, $p(y_{n+1}|F_{n})$, by sampling the following conditional distribution:

(9) $$\begin{array}{ccc} y_{n+1}^{\left[r\right]}|F_{n},\theta^{\left[r\right]} & ∼ & T_{2}^{*}\left(\mu_{n+1}^{\left[r\right]},\Sigma_{n+1}^{*\left[r\right]},\epsilon^{\left[r\right]}\right)\\ T_{2}^{*}\left(y_{n+1}^{\left[r\right]}|\mu_{n+1}^{\left[r\right]},\Sigma_{n+1}^{*\left[r\right]},\epsilon^{\left[r\right]}\right) & = & \int_{0}^{\infty }\int_{0}^{\infty }N_{2}\left(y_{n+1}^{\left[r\right]}\left|\mu_{n+1}^{\left[r\right]},\lambda_{n+1}^{-1/2}\Sigma_{n+1}^{*\left[r\right]}\lambda_{n+1}^{-1/2}\right.\right)\\ & \times & \prod_{i=1}^{2}Ga\left(\lambda_{i,n+1}\left|\frac{\nu_{i}^{\left[r\right]}}{2},\frac{\nu_{i}^{\left[r\right]}}{2}\right.\right)d\lambda_{1,n+1}d\lambda_{2,n+1}, \end{array}$$

where $\mu_{n+1}^{\left[r\right]}$ and $\Sigma_{n+1}^{*\left[r\right]}=\mathrm{diag}\left(h_{1,n+1}^{\left[r\right]},h_{2,n+1}^{\left[r\right]}\right)\Sigma_{n+1}^{\left[r\right]}$ are a conditional mean and the covariance of $p(y_{n+1}|F_{n},\theta )$ at the *r*th iteration. To assess the correctness of a VaR performance, we calculate the violation rate (VRate). The accuracy of a VaR performance is verified by recording the failure rate; that is, the violation rate:

(10) $$\begin{array}{c} \mathrm{Violation}\;\mathrm{rate}=\frac{1}{h_{0}}\sum_{t=n+1}^{n+h_{0}}I\left(r_{t}<-{\mathrm{VaR}}_{t}\right) \end{array}$$

where $h_{0}$ is the out-of-sample period size and $r_{t}$ is the return at time t. We use two tests to assess the validity of the VaR forecasts: the conditional coverage (CC) test created by [21] and the unconditional coverage (UC) test created by [22]. The CC test is conducted to investigate the null hypothesis that the violations are independently distributed, whereas the UC test is applied to determine whether the percentage of violations is equivalent to the VaR significance level.

## 5. Simulation Study

In order to access the effectiveness of the Bayesian approach, we run two simulations of the suggested models in this section. Model 1 is the bivariate HAR(1)–GJR–GARCH(1,1) model and Model 2 is the bivariate HAR(1)–QGARCH(1,1) model. Model 1 is given by:

(11) $$\begin{array}{ccc} y_{t} & = & \Phi_{0}^{\left(J_{t}\right)}+\Phi_{1}^{\left(J_{t}\right)}y_{t-1}+a_{t},\\ a_{t} & = & \mathrm{diag}\left(\sqrt{h_{1,t}},\sqrt{h_{2,t}}\right)\epsilon_{t},\;\;\epsilon_{t}∼T_{2}^{*}\left(0,\Sigma_{t},\nu \right)\\ \epsilon_{t}|\Lambda_{t} & ∼ & N_{2}\left(0,\Lambda_{t}^{-1/2}\Sigma_{t}\Lambda_{t}^{-1/2}\right), \end{array}$$

$$\begin{array}{c} \Phi_{0}^{\left(1\right)}=\left[\begin{array}{c} \phi_{10}^{\left(1\right)}\\ \phi_{20}^{\left(1\right)} \end{array}\right]=\left[\begin{array}{c} -0.10\\ -0.10 \end{array}\right],\Phi_{1}^{\left(1\right)}=\left[\begin{array}{cc} \phi_{11}^{\left(1\right)} & \phi_{12}^{\left(1\right)}\\ \phi_{21}^{\left(1\right)} & \phi_{22}^{\left(1\right)} \end{array}\right]=\left[\begin{array}{cc} 0.20 & 0.25\\ 0.25 & 0.30 \end{array}\right]\\ \;\\ \Phi_{0}^{\left(2\right)}=\left[\begin{array}{c} \phi_{10}^{\left(2\right)}\\ \phi_{20}^{\left(2\right)} \end{array}\right]=\left[\begin{array}{c} -0.08\\ -0.15 \end{array}\right]\;,\Phi_{1}^{\left(2\right)}=\left[\begin{array}{cc} \phi_{11}^{\left(2\right)} & \phi_{12}^{\left(2\right)}\\ \phi_{21}^{\left(2\right)} & \phi_{22}^{\left(2\right)} \end{array}\right]=\left[\begin{array}{cc} 0.30 & 0.35\\ 0.35 & 0.30 \end{array}\right] \end{array}$$

$$\begin{array}{ccc} \mathrm{with}\;h_{1,t} & = & \left\{\begin{array}{cc} 0.07+0.20a_{1,t-1}^{2}+0.20h_{1,t-1}+0.40I_{1,t-1}\left\{\epsilon_{t-1}<0\right\}a_{1,t-1}^{2} & \mathrm{if}J_{t}=1,\\ 0.03+0.20a_{1,t-1}^{2}+0.25h_{1,t-1}+0.55I_{1,t-1}\left\{\epsilon_{t-1}<0\right\}a_{1,t-1}^{2} & \mathrm{if}J_{t}=2, \end{array}\right.\\ h_{2,t} & = & \left\{\begin{array}{cc} 0.04+0.25a_{2,t-1}^{2}+0.10h_{2,t-1}+0.40I_{2,t-1}\left\{\epsilon_{t-1}<0\right\}a_{2,t-1}^{2} & \mathrm{if}J_{t}=1,\\ 0.02+0.30a_{2,t-1}^{2}+0.15h_{2,t-1}+0.40I_{2,t-1}\left\{\epsilon_{t-1}<0\right\}a_{2,t-1}^{2} & \mathrm{if}J_{t}=2, \end{array}\right.\\ \Sigma_{t} & = & \left\{\begin{array}{cc} \left(1-0.40-0.10\right)\Sigma^{\left(1\right)}+0.40\Sigma_{t-1}+0.10\Psi_{t-1} & \mathrm{if}J_{t}=1,\\ \left(1-0.50-0.20\right)\Sigma^{\left(2\right)}+0.50\Sigma_{t-1}+0.20\Psi_{t-1} & \mathrm{if}J_{t}=2, \end{array}\right.\\ \mathrm{where}\;J_{t} & = & \left\{\begin{array}{ccc} 1 & \mathrm{if} & y_{1,t-d}<-0.5,\\ J_{t-1} & \mathrm{if} & -0.45\leq y_{1,t-d}\leq 0.1,\\ 2 & \mathrm{if} & y_{1,t-d}>0.1, \end{array}\right.\\ \mathrm{and}\;\Sigma^{\left(1\right)} & = & \left[\begin{array}{cc} 1 & 0.65\\ 0.65 & 1 \end{array}\right],\;\Sigma^{\left(2\right)}=\left[\begin{array}{cc} 1 & 0.8\\ 0.8 & 1 \end{array}\right],\;\nu =\left[\begin{array}{c} \nu_{1}\\ \nu_{2} \end{array}\right]=\left[\begin{array}{c} 8.0\\ 10.0 \end{array}\right],\;\mathrm{and}\;d=1. \end{array}$$

Model 2 is described as follows:

(12) $$\begin{array}{ccc} y_{t} & = & \Phi_{0}^{\left(J_{t}\right)}+\Phi_{1}^{\left(J_{t}\right)}y_{t-1}+a_{t},\\ a_{t} & = & \mathrm{diag}\left(\sqrt{h_{1,t}},\sqrt{h_{2,t}}\right)\epsilon_{t},\;\;\epsilon_{t}∼T_{2}^{*}\left(0,\Sigma_{t},\nu \right) \end{array}$$

$$\begin{array}{c} \Phi_{0}^{\left(1\right)}=\left[\begin{array}{c} \phi_{10}^{\left(1\right)}\\ \phi_{20}^{\left(1\right)} \end{array}\right]=\left[\begin{array}{c} -0.10\\ -0.08 \end{array}\right],\Phi_{1}^{\left(1\right)}=\left[\begin{array}{cc} \phi_{11}^{\left(1\right)} & \phi_{12}^{\left(1\right)}\\ \phi_{21}^{\left(1\right)} & \phi_{22}^{\left(1\right)} \end{array}\right]=\left[\begin{array}{cc} 0.32 & 0.30\\ 0.37 & 0.35 \end{array}\right]\\ \;\\ \Phi_{0}^{\left(2\right)}=\left[\begin{array}{c} \phi_{10}^{\left(2\right)}\\ \phi_{20}^{\left(2\right)} \end{array}\right]=\left[\begin{array}{c} -0.08\\ -0.08 \end{array}\right]\;,\Phi_{1}^{\left(2\right)}=\left[\begin{array}{cc} \phi_{11}^{\left(2\right)} & \phi_{12}^{\left(2\right)}\\ \phi_{21}^{\left(2\right)} & \phi_{22}^{\left(2\right)} \end{array}\right]=\left[\begin{array}{cc} 0.35 & 0.30\\ 0.33 & 0.37 \end{array}\right] \end{array}$$

$$\begin{array}{ccc} \mathrm{with}\;h_{1t} & = & \left\{\begin{array}{cc} 0.07+0.20a_{1,t-1}^{2}+0.10h_{1,t-1}+0.40a_{1,t-1} & \mathrm{if}J_{t}=1,\\ 0.03+0.30a_{1,t-1}^{2}+0.10h_{1,t-1}+0.35a_{1,t-1} & \mathrm{if}J_{t}=2, \end{array}\right.\\ h_{2t} & = & \left\{\begin{array}{cc} 0.04+0.40a_{2,t-1}^{2}+0.05h_{2,t-1}+0.30a_{2,t-1} & \mathrm{if}J_{t}=1,\\ 0.02+0.30a_{2,t-1}^{2}+0.10h_{2,t-1}+0.20a_{2,t-1} & \mathrm{if}J_{t}=2, \end{array}\right.\\ \Sigma_{t} & = & \left\{\begin{array}{cc} \left(1-0.40-0.35\right)\Sigma^{\left(1\right)}+0.40\Sigma_{t-1}+0.35\Psi_{t-1} & \mathrm{if}J_{t}=1,\\ \left(1-0.55-0.15\right)\Sigma^{\left(2\right)}+0.55\Sigma_{t-1}+0.15\Psi_{t-1} & \mathrm{if}J_{t}=2, \end{array}\right.\\ \mathrm{where}\;J_{t} & = & \left\{\begin{array}{ccc} 1 & \mathrm{if} & y_{1,t-d}<-0.45,\\ J_{t-1} & \mathrm{if} & -0.45\leq y_{1,t-d}\leq 0.1,\\ 2 & \mathrm{if} & y_{1,t-d}>0.1, \end{array}\right.\\ \mathrm{and}\;\Sigma^{\left(1\right)} & = & \left[\begin{array}{cc} 1 & 0.5\\ 0.5 & 1 \end{array}\right],\;\Sigma^{\left(2\right)}=\left[\begin{array}{cc} 1 & 0.85\\ 0.85 & 1 \end{array}\right],\;\nu =\left[\begin{array}{c} \nu_{1}\\ \nu_{2} \end{array}\right]=\left[\begin{array}{c} 8.0\\ 10.0 \end{array}\right],\;\mathrm{and}\;d=1. \end{array}$$

Models 1 and 2 are created utilizing the actual values shown in Table 1 and Table 2. For each time series, we set up the sample size *n* = 2000. We carry out *N* = 30,000 MCMC iterations and discard the first *M* = 10,000 burn-in iterates. For the hyper–parameters, we choose the initial values for all parameters of the investigated model to be $\mu_{0i}=0$, $\mathrm{diag}(\Sigma_{0i})=0.1$, $l_{1}={℘}_{20}$, $u_{2}={℘}_{80}$, $c_{L}=2$, $c_{U}=60$, and $d_{0}=3$.

**Figure 1:**
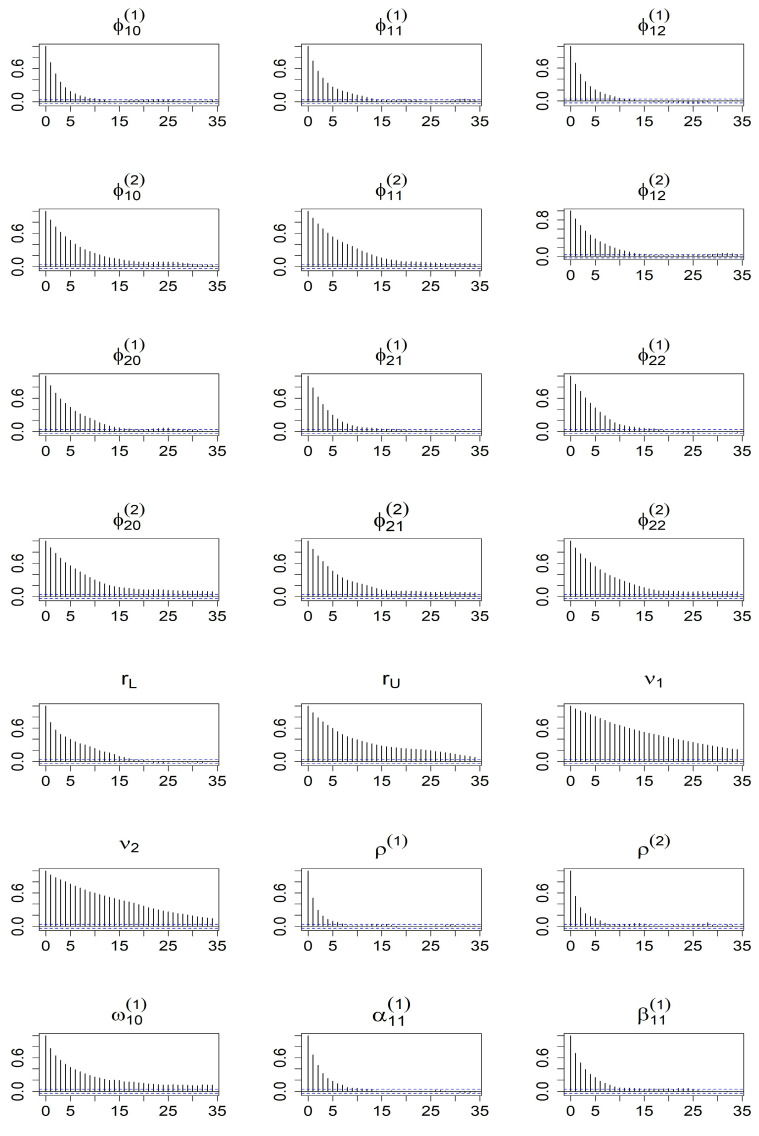
ACF plots of after burn-in MCMC iterations for all parameters from the BHAR(1)–QGARCH(1,1) model.

**Figure 2:**
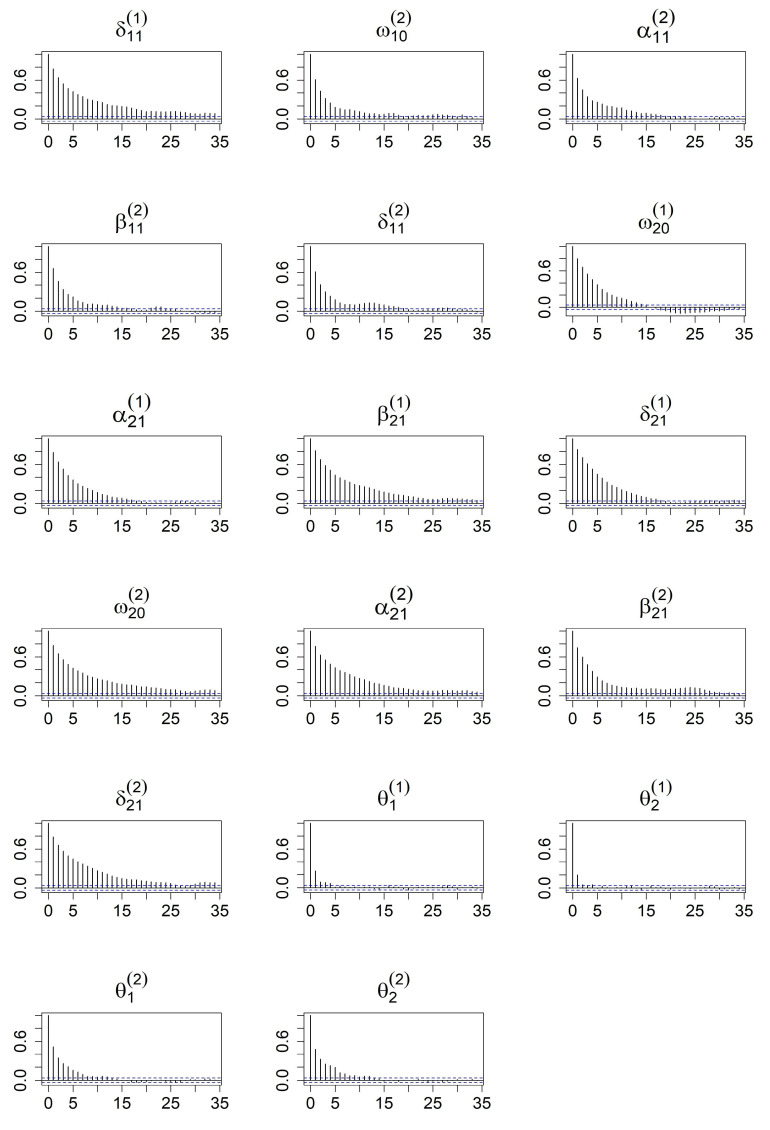
ACF plots of after burn-in MCMC iterations for all parameters from the BHAR(1)–QGARCH(1,1) model.

The results for the parameter estimates of the simulation study are shown in Table 1 and Table 2. The tables present the posterior means, medians, standard deviations, and 95% credible ranges for Models 1–2 over the 200 replications. We observe that the 95% credible interval contains the corresponding true value for each parameter. The posterior means and medians in each case are fairly close to the true parameter values. The posterior modes of lag *d* are demonstrated, and it can be explained that the posterior mode of *d* provides a reliable estimate of the delay parameter because the posterior probability for d=1 is nearly equal to one. To check the convergence of MCMC, we examine the ACF plots of all coefficients. For compactness, we present only the autocorrelation function (ACF) plots based on Model 2, omitting the ACF plots of Model 1 to conserve space. Figure 1 and Figure 2 provide additional evidence supporting the convergence of the MCMC algorithm. Based on these diagnostic checks, we conclude that the proposed models are well suited for implementation within the Bayesian framework.

## 6. Emperical Study

The empirical analysis in this study is based on the daily closing prices of four major financial indices: the S&P500, Bank of America (BAC), Intercontinental Exchange (ICE), and Goldman Sachs (GS). The data span from 4 January 2006, to 30 December 2021, encompassing a total of 4026 trading days. These data were retrieved from Yahoo Finance, a widely recognized source for historical market data. To construct the return series, we compute the continuously compounded returns (log-returns) using the formula $y_{t}=\log\left(p_{t}\right)-\log\left(p_{t-1}\right)$, where $p_{t}$ denotes the asset’s closing price at time *t*.

**Figure 3:**
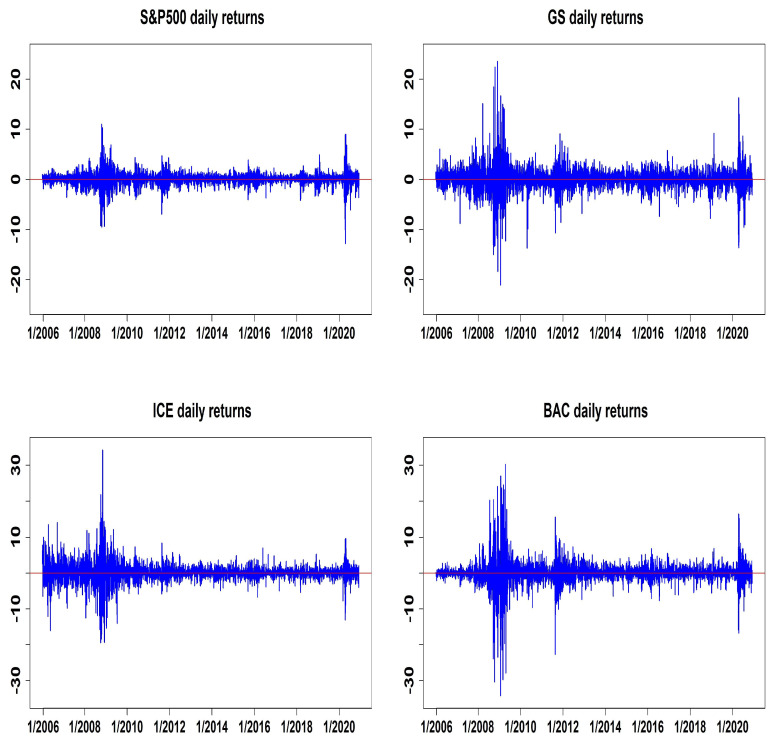
Time series plots of S&P 500, GS, ICE, and BAC daily returns.

Table 3 defines three target datasets: DS1 {S&P500, GS}, DS2 {S&P500, ICE}, and DS3 {S&P500, BAC}. It also presents the descriptive statistics of the corresponding return series, along with the results of two multivariate normality tests: Mardia’s test and the Henze–Zirkler test (see [23,24]). The return distributions are clearly skewed and have high kurtosis, in particular, showing strong positive skewness. Due to the noticeable asymmetry and the presence of heavy tails in the return data, we recommend using asymmetric models with fat-tailed multivariate error distributions instead of models that assume multivariate normal errors. Figure 3 presents the time series plot of daily returns for the selected financial assets. As shown, the sample period spans several significant market events, notably the Global Financial Crisis, which officially began on 15 September 2008, following the bankruptcy of Lehman Brothers. For the purpose of estimation and out-of-sample evaluation, the dataset is divided into two distinct sub-periods. The first segment, consisting of 3726 daily observations, is used to estimate the model parameters. The remaining 300 observations are reserved for out-of-sample forecasting and performance assessment.

This section’s hyper-parameters correspond to those in the simulation study. Table 4, Table 5, Table 6 and Table 7 present a summary of the Bayesian estimates for three datasets for the BHAR(1)–GJR–GARCH(1,1) and the BHAR(1)–QGARCH(1,1) models. The significant value of $\phi_{12}^{\left(1\right)}$ in Table 4 and Table 6 indicates that the performance of the previous day’s return of Goldman Sachs stock has a considerable negative impact on the S&P 500’s returns in the lower regimes. We can see that the parameter estimates for $\phi_{12}^{\left(1\right)}$, $\phi_{12}^{\left(2\right)}$, $\phi_{21}^{\left(1\right)}$, and $\phi_{21}^{\left(2\right)}$ are identical in both fitted models when we look at datasets DS2 and DS3 in Table 5 and Table 7. To assess the validity of the proposed models, we further employ the Geweke convergence diagnostic (see [25]). The *p*-values reported in Table 8 and Table 9 suggest that the MCMC chains generated from the models have converged. As there is no statistical evidence of non-convergence, we conclude that the proposed models are appropriately specified and reliable for inference.

**Figure 4:**
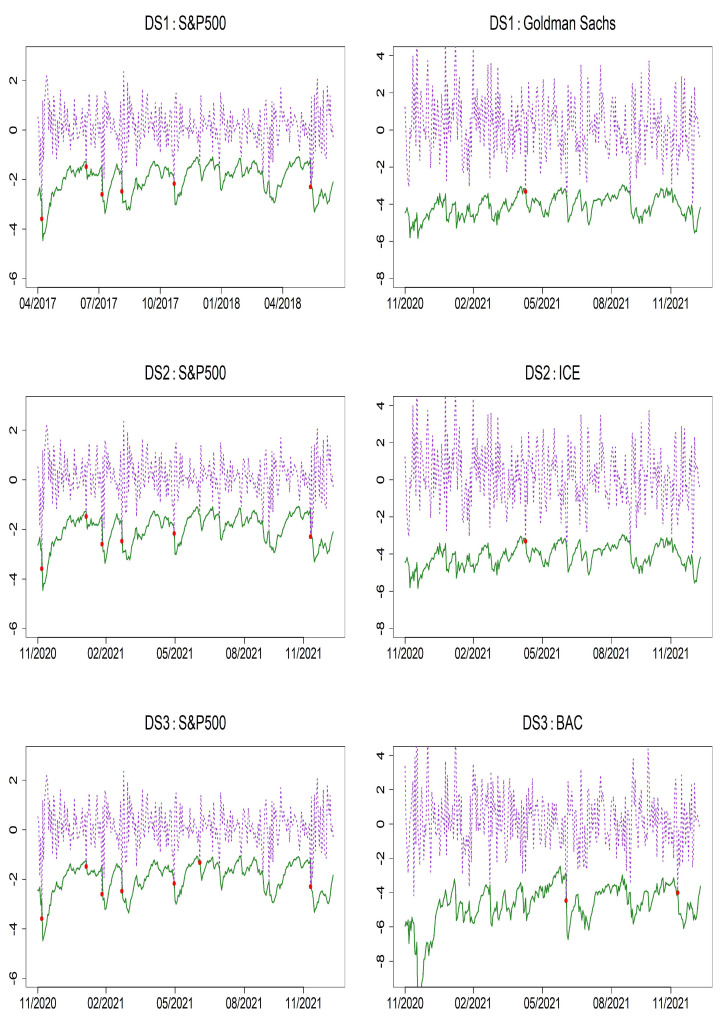
VaR forecast performance at the 1% level over 300 out-of-sample periods using the BHAR(1)–GJR–GARCH(1,1) model. Solid line: 1% VaR forecasts; dashed line: daily returns; red dots: VaR violations.

**Figure 5:**
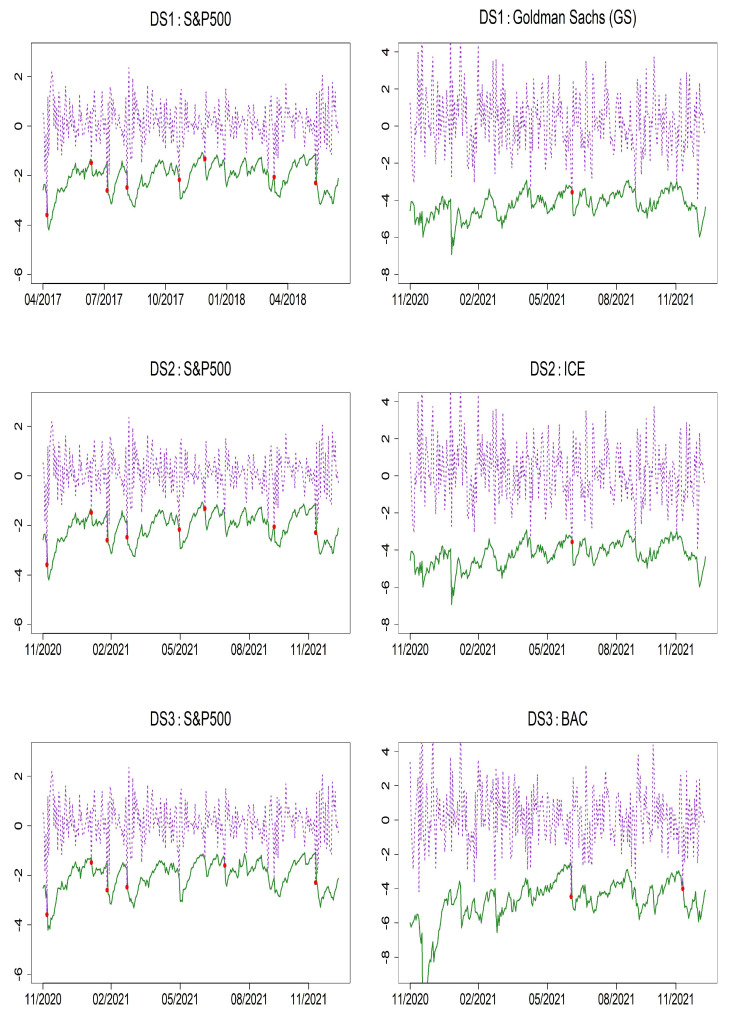
VaR forecast performance at the 1% level over 300 out-of-sample periods using the BHAR(1)–-QGARCH(1,1) model. Solid line: 1% VaR forecasts; dashed line: daily returns; red dots: VaR violations.

To evaluate the accuracy of the models using Value-at-Risk (VaR), we present VaR forecasts along with the results of VaR backtesting at the 1% and 5% significance levels. Specifically, Table 10 and Table 11 report the *p*-values of the Unconditional Coverage (UC) and Conditional Coverage (CC) tests for the two proposed models: bivariate HAR(1)–GJR–GARCH(1,1) and HAR(1)–QGARCH(1,1) as well as the benchmark bivariate HAR(1)–GARCH(1,1) model. When evaluating DS1, DS2, and DS3 across the three models, the violation rates (VRate) for the S&P 500 tend to be significantly higher than the nominal 1% level, suggesting a slight underestimation of tail risk. In contrast, the VRates for Bank of America (BAC), Intercontinental Exchange (ICE), and Goldman Sachs (GS) indicate a tendency toward risk overestimation. Nevertheless, the backtesting results show that all three models perform adequately as risk models. At the 5% significance level, both the proposed models and the benchmark BHAR(1)–GARCH(1,1) model yield UC and CC test *p*-values above 5%, indicating no statistical evidence of model misspecification. These findings confirm that the proposed models deliver reliable and independent risk forecasts. Figure 4 and Figure 5 display the VaR forecasts based on the bivariate HAR(1)–GJR–GARCH(1,1) and HAR(1)–QGARCH(1,1) models, which clearly show that the models are capable of identifying volatility spikes in returns, despite infrequent violations of the forecast bounds.

To understand how well the proposed models can capture the expected shortfall movement, we present the backtesting measures of the MES forecasts proposed by the authors of [26] in Table 12. The model with the smallest values in the boxes is the best. These findings indicate that the proposed models are the best.

## 7. Conclusions

This paper investigates the MHAR–GJR–GARCH and MHAR–QGARCH models by incorporating asymmetric volatility dynamics, conditional correlations, and a hysteresis variable to control regime switching and dynamic delays. Bayesian inference is employed for efficient estimation of the model parameters. A comparative analysis of backtesting results for the VaR and MES forecasts is conducted. We also include the benchmark model MHAR–GARCH with adapted multivariate Student’s-t errors and compare backtesting measures of the Value-at-Risk (VaR) and Marginal Expected Shortfall (MES) forecasts. The backtesting measures indicate that, in general, the proposed models demonstrate reliable capabilities in capturing tail risk behavior and delivering accurate risk predictions. Notably, the proposed models deliver significantly improved performance over the original MHAR–GARCH errors model, particularly in capturing asymmetric tail risks and providing more accurate risk forecasts. An interesting direction for future research involves incorporating entropy-based measures, such as those discussed in [27,28], as complementary indicators of volatility and structural regime shifts. As entropy captures informational complexity and system disorder, it may enhance regime detection when combined with hysteresis-based models.

## Figures and Tables

**Table 1:** Simulation results of the BHAR(1)–GJR–GARCH(1,1) model obtained from 200 replications.

Parameter	True	Mean	Med	Std	2.5%	97.5%	Coverage
$\phi_{10}^{\left(1\right)}$	−0.10	−0.1022	−0.1023	0.0280	−0.1573	−0.0472	94.00
$\phi_{20}^{\left(1\right)}$	−0.10	−0.1014	−0.1015	0.0185	−0.1375	−0.0650	98.00
$\phi_{11}^{\left(1\right)}$	0.20	0.1992	0.1992	0.0482	0.1048	0.2936	95.50
$\phi_{12}^{\left(1\right)}$	0.25	0.2465	0.2465	0.0440	0.1602	0.3330	95.50
$\phi_{21}^{\left(1\right)}$	0.25	0.2510	0.2511	0.0215	0.2089	0.2932	96.00
$\phi_{22}^{\left(1\right)}$	0.30	0.2959	0.2960	0.0328	0.2312	0.3601	97.00
$\phi_{10}^{\left(2\right)}$	−0.08	−0.0811	−0.0811	0.0119	−0.1045	−0.0579	94.50
$\phi_{20}^{\left(2\right)}$	−0.15	−0.1512	−0.1512	0.0081	−0.1671	−0.1354	92.00
$\phi_{11}^{\left(2\right)}$	0.30	0.3005	0.3005	0.0347	0.2323	0.3688	95.50
$\phi_{12}^{\left(2\right)}$	0.35	0.3472	0.3472	0.0344	0.2797	0.4149	95.00
$\phi_{21}^{\left(2\right)}$	0.35	0.3514	0.3514	0.0162	0.3197	0.3832	94.00
$\phi_{22}^{\left(2\right)}$	0.30	0.2972	0.2972	0.0234	0.2513	0.3432	95.00
$r_{L}$	−0.50	−0.4989	−0.4988	0.0184	−0.5334	−0.4640	94.50
$r_{U}$	0.10	0.0885	0.0890	0.0324	0.0266	0.1503	92.50
$\nu_{1}$	8.00	9.1324	8.9642	1.4995	6.6907	12.5705	97.50
$\nu_{2}$	10.00	10.1588	9.9447	1.7664	7.3307	14.2193	98.50
$\rho^{\left(1\right)}$	0.65	0.6460	0.6483	0.0323	0.5758	0.7021	97.50
$\rho^{\left(2\right)}$	0.80	0.7990	0.7990	0.0295	0.7414	0.8572	95.50
*d*	1.00	1.0000	1.0000	0.0204	1.0000	1.0000	100.00
$\omega_{10}^{\left(1\right)}$	0.07	0.0782	0.0775	0.0144	0.0521	0.1088	89.50
$\alpha_{11}^{\left(1\right)}$	0.20	0.2139	0.2091	0.1148	0.0218	0.4385	100.00
$\beta_{11}^{\left(1\right)}$	0.20	0.2191	0.2167	0.1143	0.0243	0.4388	100.00
$\delta_{11}^{\left(1\right)}$	0.40	0.3821	0.3819	0.0613	0.2620	0.5020	91.00
$\omega_{10}^{\left(2\right)}$	0.03	0.0349	0.0345	0.0073	0.0217	0.0506	91.00
$\alpha_{11}^{\left(2\right)}$	0.20	0.2147	0.2131	0.0356	0.1502	0.2899	96.50
$\beta_{11}^{\left(2\right)}$	0.25	0.2792	0.2754	0.1150	0.0721	0.5080	97.00
$\delta_{11}^{\left(2\right)}$	0.55	0.5240	0.5248	0.0492	0.4245	0.6184	93.00
$\omega_{20}^{\left(1\right)}$	0.04	0.0382	0.0379	0.0068	0.0259	0.0524	93.00
$\alpha_{21}^{\left(1\right)}$	0.25	0.2458	0.2439	0.0682	0.1190	0.3837	97.00
$\beta_{21}^{\left(1\right)}$	0.10	0.1262	0.1196	0.0694	0.0163	0.2754	97.50
$\delta_{21}^{\left(1\right)}$	0.40	0.3781	0.3781	0.0677	0.2450	0.5107	96.00
$\omega_{20}^{\left(2\right)}$	0.02	0.0218	0.0216	0.0039	0.0147	0.0298	94.00
$\alpha_{21}^{\left(2\right)}$	0.30	0.3063	0.3044	0.0441	0.2253	0.3971	97.00
$\beta_{21}^{\left(2\right)}$	0.15	0.1830	0.1769	0.0830	0.0430	0.3542	95.00
$\delta_{21}^{\left(2\right)}$	0.40	0.3808	0.3809	0.0525	0.2781	0.4824	94.00
$\theta_{1}^{\left(1\right)}$	0.40	0.3915	0.3986	0.1819	0.0710	0.7004	97.00
$\theta_{2}^{\left(1\right)}$	0.10	0.1011	0.0968	0.0476	0.0258	0.1938	96.50
$\theta_{1}^{\left(2\right)}$	0.50	0.4615	0.4672	0.1083	0.2370	0.6571	96.50
$\theta_{2}^{\left(2\right)}$	0.20	0.2092	0.2065	0.0412	0.1364	0.2969	95.50

**Table 2:** Simulation results of the BHAR(1)–QGARCH(1,1) model obtained from 200 replications.

Parameter	True	Mean	Med	Std	2.5%	97.5%	Coverage
$\phi_{10}^{\left(1\right)}$	−0.10	−0.1003	−0.1002	0.0203	−0.1404	−0.0606	94.00
$\phi_{20}^{\left(1\right)}$	−0.08	−0.0792	−0.0792	0.0153	−0.1093	−0.0493	93.50
$\phi_{11}^{\left(1\right)}$	0.32	0.3185	0.3186	0.0351	0.2494	0.3871	94.00
$\phi_{12}^{\left(1\right)}$	0.30	0.2973	0.2972	0.0292	0.2401	0.3548	97.00
$\phi_{21}^{\left(1\right)}$	0.37	0.3717	0.3717	0.0217	0.3290	0.4143	94.50
$\phi_{22}^{\left(1\right)}$	0.35	0.3467	0.3467	0.0250	0.2976	0.3958	95.00
$\phi_{10}^{\left(2\right)}$	−0.08	−0.0808	−0.0808	0.0108	−0.1021	−0.0595	96.50
$\phi_{20}^{\left(2\right)}$	−0.08	−0.0802	−0.0802	0.0070	−0.0940	−0.0665	95.50
$\phi_{11}^{\left(2\right)}$	0.35	0.3427	0.3427	0.0394	0.2652	0.4197	94.00
$\phi_{12}^{\left(2\right)}$	0.30	0.3027	0.3027	0.0372	0.2295	0.3759	95.00
$\phi_{21}^{\left(2\right)}$	0.33	0.3290	0.3290	0.0183	0.2930	0.3647	94.00
$\phi_{22}^{\left(2\right)}$	0.37	0.3666	0.3667	0.0235	0.3204	0.4127	95.00
$r_{L}$	−0.45	−0.4501	−0.4503	0.0069	−0.4626	−0.4370	93.00
$r_{U}$	0.10	0.0970	0.0973	0.0113	0.0750	0.1170	93.00
$\nu_{1}$	8.00	9.2129	9.0257	1.5569	6.7229	12.8309	93.50
$\nu_{2}$	10.00	10.2736	10.0551	1.8017	7.3914	14.4714	99.50
$\rho^{\left(1\right)}$	0.50	0.4951	0.4994	0.0563	0.3723	0.5928	92.50
$\rho^{\left(2\right)}$	0.85	0.8472	0.8479	0.0257	0.7948	0.8958	94.50
*d*	1.00	1.0000	1.0000	0.0152	1.0000	1.0000	100.00
$\omega_{10}^{\left(1\right)}$	0.07	0.0784	0.0778	0.0124	0.0557	0.1040	91.50
$\alpha_{11}^{\left(1\right)}$	0.20	0.2235	0.2213	0.0467	0.1386	0.3220	95.00
$\beta_{11}^{\left(1\right)}$	0.10	0.1107	0.1098	0.0289	0.0566	0.1704	96.00
$\delta_{11}^{\left(1\right)}$	0.40	0.3613	0.3628	0.0832	0.1955	0.5205	95.50
$\omega_{10}^{\left(2\right)}$	0.03	0.0343	0.0340	0.0060	0.0234	0.0470	92.00
$\alpha_{11}^{\left(2\right)}$	0.30	0.3306	0.3282	0.0558	0.2291	0.4473	94.00
$\beta_{11}^{\left(2\right)}$	0.10	0.1030	0.1032	0.0236	0.0555	0.1490	96.50
$\delta_{11}^{\left(2\right)}$	0.35	0.3289	0.3285	0.0527	0.2268	0.4339	94.00
$\omega_{20}^{\left(1\right)}$	0.04	0.0371	0.0369	0.0051	0.0276	0.0478	95.00
$\alpha_{21}^{\left(1\right)}$	0.40	0.4115	0.4095	0.0555	0.3089	0.5284	94.50
$\beta_{21}^{\left(1\right)}$	0.05	0.0533	0.0525	0.0192	0.0180	0.0932	95.00
$\delta_{21}^{\left(1\right)}$	0.30	0.2855	0.2850	0.0618	0.1664	0.4087	95.00
$\omega_{20}^{\left(2\right)}$	0.02	0.0212	0.0211	0.0026	0.0163	0.0267	94.50
$\alpha_{21}^{\left(2\right)}$	0.30	0.3212	0.3197	0.0449	0.2387	0.4122	95.50
$\beta_{21}^{\left(2\right)}$	0.10	0.1032	0.1031	0.0138	0.0767	0.1308	93.00
$\delta_{21}^{\left(2\right)}$	0.20	0.1904	0.1892	0.0410	0.1129	0.2733	96.00
$\theta_{1}^{\left(1\right)}$	0.40	0.3810	0.3847	0.1001	0.1753	0.5660	94.00
$\theta_{2}^{\left(1\right)}$	0.35	0.3582	0.3561	0.0563	0.2543	0.4744	96.00
$\theta_{1}^{\left(2\right)}$	0.55	0.5157	0.5244	0.0928	0.3104	0.6746	96.50
$\theta_{2}^{\left(2\right)}$	0.15	0.1573	0.1531	0.0427	0.0850	0.2535	98.00

**Table 3:** Summary statistics and multivariate normality tests.

Data	Mean	Std	Min	Max	Skewness	Kurtosis	MVN Tests *	
							**(*p*-Value)**	
							**Mardia**	** Henze–Zirkler **
S&P500	0.033	1.256	−12.765	10.957	−0.568	16.737		
GS	0.033	2.320	−21.022	23.482	0.188	18.086		
ICE	0.075	2.578	−19.501	34.217	0.205	20.699		
BAC	0.007	3.165	−34.206	30.210	−0.319	26.645		
S&P500 vs. GS							<0.001	<0.001
S&P 500 vs. ICE							<0.001	<0.001
S&P 500 vs. BAC							<0.001	<0.001

∗: “MVN” stands for multivariate normality.

**Table 4:** Estimation results, including posterior means, medians, standard deviations, and 95% Bayes credible intervals of dataset DS1 {S&P 500, GS}, based on the BHAR(1)–GJR–GARCH(1,1) model.

Parameter	Mean	Med	Std	2.5%	97.5%
$\phi_{10}^{\left(1\right)}$	0.0453	0.0454	0.0255	−0.0043	0.0967
$\phi_{20}^{\left(1\right)}$	0.0525	0.0538	0.0520	−0.0580	0.1502
$\phi_{11}^{\left(1\right)}$	−0.0914	−0.0921	0.0375	−0.1624	−0.0205
$\phi_{12}^{\left(1\right)}$	−0.0023	−0.0017	0.0178	−0.0371	0.0323
$\phi_{21}^{\left(1\right)}$	−0.0274	−0.0284	0.0649	−0.1506	0.0972
$\phi_{22}^{\left(1\right)}$	−0.0198	−0.0204	0.0353	−0.0880	0.0516
$\phi_{10}^{\left(2\right)}$	0.0408	0.0405	0.0137	0.0147	0.0680
$\phi_{20}^{\left(2\right)}$	0.0010	−0.0005	0.0340	−0.0648	0.0717
$\phi_{11}^{\left(2\right)}$	0.0054	0.0050	0.0283	−0.0500	0.0608
$\phi_{12}^{\left(2\right)}$	−0.0265	−0.0267	0.0124	−0.0499	−0.0024
$\phi_{21}^{\left(2\right)}$	0.0339	0.0321	0.0575	−0.0792	0.1423
$\phi_{22}^{\left(2\right)}$	−0.0421	−0.0411	0.0287	−0.1000	0.0111
$r_{L}$	−0.4935	−0.4744	0.0386	−0.5667	−0.4502
$r_{U}$	0.6388	0.6497	0.0295	0.5541	0.6814
$\nu_{1}$	8.8291	8.7186	0.9056	7.2395	10.9435
$\nu_{2}$	7.4454	7.4002	0.7572	6.1675	9.1533
$\rho^{\left(1\right)}$	0.8766	0.8765	0.0192	0.8393	0.9150
$\rho^{\left(2\right)}$	0.6681	0.6699	0.0318	0.6014	0.7265
*d*	1.0000	1.0000	0.0318	1.0000	1.0000
$\omega_{10}^{\left(1\right)}$	0.0247	0.0243	0.0042	0.0170	0.0341
$\alpha_{11}^{\left(1\right)}$	0.0085	0.0082	0.0049	0.0009	0.0188
$\beta_{11}^{\left(1\right)}$	0.1155	0.1153	0.0078	0.1007	0.1299
$\delta_{11}^{\left(1\right)}$	0.9285	0.9296	0.0073	0.9115	0.9389
$\omega_{10}^{\left(2\right)}$	0.0179	0.0178	0.0024	0.0135	0.0228
$\alpha_{11}^{\left(2\right)}$	0.0223	0.0222	0.0053	0.0117	0.0328
$\beta_{11}^{\left(2\right)}$	0.2750	0.2747	0.0137	0.2483	0.3006
$\delta_{11}^{\left(2\right)}$	0.8189	0.8195	0.0114	0.7959	0.8404
$\omega_{10}^{\left(2\right)}$	0.0804	0.0796	0.0158	0.0525	0.1140
$\alpha_{11}^{\left(2\right)}$	0.0268	0.0266	0.0086	0.0101	0.0435
$\beta_{11}^{\left(2\right)}$	0.0532	0.0528	0.0082	0.0370	0.0699
$\delta_{11}^{\left(2\right)}$	0.9353	0.9363	0.0112	0.9108	0.9549
$\omega_{20}^{\left(2\right)}$	0.0852	0.0850	0.0154	0.0565	0.1159
$\alpha_{21}^{\left(2\right)}$	0.0397	0.0396	0.0052	0.0298	0.0503
$\beta_{21}^{\left(2\right)}$	0.0449	0.0452	0.0107	0.0240	0.0654
$\delta_{21}^{\left(2\right)}$	0.8482	0.8483	0.0139	0.8207	0.8737
$\theta_{1}^{\left(1\right)}$	0.8058	0.8060	0.0200	0.7676	0.8446
$\theta_{2}^{\left(1\right)}$	0.0325	0.0325	0.0030	0.0266	0.0383
$\theta_{1}^{\left(2\right)}$	0.8742	0.8744	0.0162	0.8428	0.9056
$\theta_{2}^{\left(2\right)}$	0.0428	0.0427	0.0032	0.0366	0.0491

**Table 5:** Estimation results, including posterior means and 95% Bayes credible intervals of datasets DS2 and DS3, based on the BHAR(1)–GJR–GARCH(1,1) model.

Parameter	DS2				DS3		
	Mean	2.5%	97.5%		Mean	2.5%	97.5%
$\phi_{10}^{\left(1\right)}$	0.0871	0.0325	0.1398		0.0598	0.0081	0.1094
$\phi_{20}^{\left(1\right)}$	0.0742	−0.0073	0.1589		0.0017	−0.0931	0.0911
$\phi_{11}^{\left(1\right)}$	−0.0331	−0.0915	0.0291		−0.1103	−0.1858	−0.0376
$\phi_{12}^{\left(1\right)}$	−0.0299	−0.0563	−0.0035		0.0115	−0.0181	0.0395
$\phi_{21}^{\left(1\right)}$	−0.1255	−0.2212	−0.0305		−0.2095	−0.3419	−0.0754
$\phi_{22}^{\left(1\right)}$	−0.0393	−0.0947	0.0168		0.0721	0.0058	0.1372
$\phi_{10}^{\left(2\right)}$	0.0514	0.0218	0.0787		0.0471	0.0203	0.0728
$\phi_{20}^{\left(2\right)}$	0.0270	−0.0346	0.0860		0.0342	−0.0222	0.0882
$\phi_{11}^{\left(2\right)}$	−0.0372	−0.0857	0.0130		−0.0299	−0.0829	0.0255
$\phi_{12}^{\left(2\right)}$	−0.0094	−0.0241	0.0043		−0.0155	−0.0336	0.0018
$\phi_{21}^{\left(2\right)}$	−0.0181	−0.1160	0.0778		−0.1706	−0.2781	−0.0684
$\phi_{22}^{\left(2\right)}$	−0.0553	−0.1000	−0.0091		0.0213	−0.0304	0.0708
$r_{L}$	−0.5351	−0.5778	−0.4536		−0.5601	−0.5769	−0.5315
$r_{U}$	0.6238	0.5814	0.6569		0.6208	0.5852	0.6668
$\nu_{1}$	6.8541	5.6768	8.3212		8.9290	7.2431	10.8435
$\nu_{2}$	5.1780	4.4976	6.0264		6.1178	5.2628	7.0598
$\rho^{\left(1\right)}$	0.8877	0.8006	0.9790		0.8202	0.7953	0.8431
$\rho^{\left(2\right)}$	0.2767	0.1298	0.3908		0.5443	0.4328	0.6347
*d*	1.0000	1.0000	1.0000		1.0000	1.0000	1.0000
$\omega_{10}^{\left(1\right)}$	0.0210	0.0145	0.0312		0.0276	0.0174	0.0395
$\alpha_{11}^{\left(1\right)}$	0.0115	0.0018	0.0233		0.0148	0.0017	0.0302
$\beta_{11}^{\left(1\right)}$	0.0989	0.0840	0.1132		0.1115	0.0978	0.1253
$\delta_{11}^{\left(1\right)}$	0.9337	0.9178	0.9441		0.9221	0.9013	0.9384
$\omega_{10}^{\left(2\right)}$	0.0142	0.0100	0.0188		0.0169	0.0125	0.0216
$\alpha_{11}^{\left(2\right)}$	0.0164	0.0040	0.0305		0.0224	0.0124	0.0336
$\beta_{11}^{\left(2\right)}$	0.2234	0.1814	0.2657		0.2727	0.2426	0.2999
$\delta_{11}^{\left(2\right)}$	0.8368	0.8118	0.8592		0.8261	0.8047	0.8456
$\omega_{20}^{\left(1\right)}$	0.0424	0.0202	0.0703		0.1096	0.0734	0.1484
$\alpha_{21}^{\left(1\right)}$	0.0313	0.0118	0.0512		0.0653	0.0401	0.0927
$\beta_{21}^{\left(1\right)}$	0.0481	0.0309	0.0654		0.0472	0.0247	0.0692
$\delta_{21}^{\left(1\right)}$	0.9356	0.9073	0.9562		0.8704	0.8372	0.8974
$\omega_{20}^{\left(2\right)}$	0.0366	0.0206	0.0550		0.0165	0.0020	0.0358
$\alpha_{21}^{\left(2\right)}$	0.0533	0.0424	0.0653		0.0540	0.0423	0.0667
$\beta_{21}^{\left(2\right)}$	0.0381	0.0242	0.0525		0.0996	0.0756	0.1234
$\delta_{21}^{\left(2\right)}$	0.8506	0.8217	0.8776		0.8644	0.8381	0.8857
$\theta_{1}^{\left(1\right)}$	0.8311	0.7798	0.8711		0.3327	0.2778	0.3865
$\theta_{2}^{\left(1\right)}$	0.0599	0.0521	0.0683		0.1297	0.1155	0.1438
$\theta_{1}^{\left(2\right)}$	0.9266	0.9114	0.9419		0.9137	0.8977	0.9292
$\theta_{2}^{\left(2\right)}$	0.0254	0.0211	0.0297		0.0340	0.0294	0.0384

**Table 6:** Results of estimation of the BHAR(1)–QGARCH(1,1) model are shown, including posterior means, medians, standard deviations, and 95% Bayes credible intervals of the dataset DS1.

Parameter	Mean	Med	Std	2.5%	97.5%
$\phi_{10}^{\left(1\right)}$	0.0139	0.0128	0.0328	−0.0493	0.0796
$\phi_{20}^{\left(1\right)}$	−0.0248	−0.0279	0.0584	−0.1333	0.0933
$\phi_{11}^{\left(1\right)}$	−0.0638	−0.0663	0.0454	−0.1448	0.0290
$\phi_{12}^{\left(1\right)}$	−0.0370	−0.0370	0.0170	−0.0699	−0.0039
$\phi_{21}^{\left(1\right)}$	−0.0100	−0.0138	0.0797	−0.1648	0.1542
$\phi_{22}^{\left(1\right)}$	−0.0657	−0.0651	0.0375	−0.1386	0.0053
$\phi_{10}^{\left(2\right)}$	0.0338	0.0336	0.0153	0.0052	0.0658
$\phi_{20}^{\left(2\right)}$	0.0179	0.0191	0.0347	−0.0535	0.0869
$\phi_{11}^{\left(2\right)}$	−0.0217	−0.0224	0.0298	−0.0792	0.0379
$\phi_{12}^{\left(2\right)}$	−0.0050	−0.0048	0.0122	−0.0290	0.0201
$\phi_{21}^{\left(2\right)}$	−0.0261	−0.0244	0.0592	−0.1475	0.0836
$\phi_{22}^{\left(2\right)}$	−0.0072	−0.0071	0.0274	−0.0617	0.0481
$r_{L}$	−0.1680	−0.1595	0.0222	−0.2108	−0.1405
$r_{U}$	0.0179	−0.0013	0.0449	−0.0329	0.1243
$\nu_{1}$	8.7278	8.6580	0.9039	7.0614	10.6549
$\nu_{2}$	7.3638	7.3355	0.6199	6.1993	8.6153
$\rho^{\left(1\right)}$	0.8502	0.8501	0.0134	0.8239	0.8759
$\rho^{\left(2\right)}$	0.2581	0.3011	0.2326	−0.3197	0.5698
*d*	1.0000	1.0000	0.2326	1.0000	1.0000
$\omega_{10}^{\left(1\right)}$	0.0817	0.0813	0.0043	0.0742	0.0902
$\alpha_{11}^{\left(1\right)}$	0.1243	0.1244	0.0087	0.1066	0.1415
$\beta_{11}^{\left(1\right)}$	0.0092	0.0093	0.0032	0.0026	0.0153
$\delta_{11}^{\left(1\right)}$	0.8724	0.8728	0.0096	0.8534	0.8916
$\omega_{10}^{\left(2\right)}$	0.0036	0.0035	0.0014	0.0009	0.0065
$\alpha_{11}^{\left(2\right)}$	0.0201	0.0202	0.0050	0.0106	0.0300
$\beta_{11}^{\left(2\right)}$	0.0037	0.0033	0.0025	0.0002	0.0093
$\delta_{11}^{\left(2\right)}$	0.8448	0.8448	0.0102	0.8237	0.8635
$\omega_{20}^{\left(1\right)}$	0.1986	0.1975	0.0243	0.1490	0.2492
$\alpha_{21}^{\left(1\right)}$	0.0886	0.0880	0.0086	0.0732	0.1066
$\beta_{21}^{\left(1\right)}$	0.0310	0.0309	0.0124	0.0083	0.0555
$\delta_{21}^{\left(1\right)}$	0.9002	0.9015	0.0140	0.8686	0.9248
$\omega_{20}^{\left(2\right)}$	0.0435	0.0425	0.0146	0.0150	0.0726
$\alpha_{21}^{\left(2\right)}$	0.0422	0.0420	0.0058	0.0314	0.0536
$\beta_{21}^{\left(2\right)}$	0.0123	0.0123	0.0052	0.0029	0.0232
$\delta_{21}^{\left(2\right)}$	0.8589	0.8595	0.0133	0.8316	0.8816
$\theta_{1}^{\left(1\right)}$	0.6106	0.6104	0.0155	0.5806	0.6422
$\theta_{2}^{\left(1\right)}$	0.0407	0.0407	0.0036	0.0338	0.0477
$\theta_{1}^{\left(2\right)}$	0.9163	0.9179	0.0158	0.8818	0.9403
$\theta_{2}^{\left(2\right)}$	0.0503	0.0504	0.0046	0.0425	0.0584

**Table 7:** Estimation results are shown, including posterior means and 95% Bayes credible intervals of datasets DS1, DS2, and DS3, based on the BHAR(1)–QGARCH(1,1) model.

Parameter	DS1				DS2				DS3		
	Mean	2.5%	97.5%		Mean	2.5%	97.5%		Mean	2.5%	97.5%
$\phi_{10}^{\left(1\right)}$	0.0139	0.0328	−0.0493		0.0476	−0.0161	0.1136		0.0753	0.0219	0.1301
$\phi_{20}^{\left(1\right)}$	−0.0248	0.0584	−0.1333		0.0654	−0.0425	0.1699		0.0267	−0.0725	0.1215
$\phi_{11}^{\left(1\right)}$	−0.0638	0.0454	0.0454		−0.0579	−0.1326	0.0212		−0.1049	−0.1784	−0.0299
$\phi_{12}^{\left(1\right)}$	−0.0370	0.0170	0.0170		−0.0376	−0.0600	−0.0135		0.0060	−0.0227	0.0332
$\phi_{21}^{\left(1\right)}$	−0.0100	0.0797	0.0797		−0.1247	−0.2361	−0.0108		−0.1902	−0.3188	−0.0611
$\phi_{22}^{\left(1\right)}$	−0.0657	0.0375	0.0375		−0.0581	−0.1167	0.0016		0.0550	−0.0102	0.1181
$\phi_{10}^{\left(2\right)}$	0.0338	0.0153	0.0153		0.0413	0.0084	0.0738		0.0418	0.0107	0.0692
$\phi_{20}^{\left(2\right)}$	0.0179	0.0347	0.0347		0.0046	−0.0635	0.0696		0.0309	−0.0239	0.0867
$\phi_{11}^{\left(2\right)}$	−0.0217	0.0298	0.0298		−0.0236	−0.0837	0.0270		−0.0236	−0.0732	0.0309
$\phi_{12}^{\left(2\right)}$	−0.0050	0.0122	0.0122		−0.0063	−0.0226	0.0099		−0.0117	−0.0292	0.0059
$\phi_{21}^{\left(2\right)}$	−0.0261	0.0592	0.0592		0.0158	−0.0893	0.1169		−0.1659	−0.2692	−0.0598
$\phi_{22}^{\left(2\right)}$	−0.0072	0.0274	0.0274		−0.0475	−0.0951	−0.0029		0.0314	−0.0202	0.0814
$r_{L}$	−0.1680	0.0222	0.0222		−0.2019	−0.2123	−0.1811		−0.5473	−0.5747	−0.4611
$r_{U}$	0.0179	0.0449	0.0449		0.0524	−0.0385	0.1507		0.6111	0.5527	0.6559
$\nu_{1}$	8.7278	0.9039	0.9039		6.8073	5.5809	8.3232		8.9350	7.3051	10.8176
$\nu_{2}$	7.3638	0.6199	0.6199		5.2143	4.5484	5.9952		6.0211	5.1460	7.0315
$\rho^{\left(1\right)}$	0.8502	0.0134	0.0134		0.9172	0.8269	0.9912		0.8109	0.7864	0.8350
$\rho^{\left(2\right)}$	0.2581	0.2326	0.2326		−0.4946	−0.9541	0.0104		0.4795	0.0228	0.6653
*d*	1.0000	0.2326	0.2326		1.0000	1.0000	1.0000		1.0000	1.0000	1.0000
$\omega_{10}^{\left(1\right)}$	0.0817	0.0043	0.0043		0.0604	0.0510	0.0712		0.0486	0.0357	0.0637
$\alpha_{11}^{\left(1\right)}$	0.1243	0.0087	0.0087		0.1046	0.0899	0.1219		0.1214	0.1067	0.1380
$\beta_{11}^{\left(1\right)}$	0.0092	0.0032	0.0032		0.0077	0.0006	0.0159		0.0077	0.0003	0.0228
$\delta_{11}^{\left(1\right)}$	0.8724	0.0096	0.0096		0.8916	0.8704	0.9085		0.8748	0.8573	0.8916
$\omega_{10}^{\left(2\right)}$	0.0036	0.0014	0.0014		0.0033	0.0007	0.0067		0.0194	0.0144	0.0249
$\alpha_{11}^{\left(2\right)}$	0.0201	0.0050	0.0050		0.0159	0.0050	0.0275		0.0318	0.0186	0.0472
$\beta_{11}^{\left(2\right)}$	0.0037	0.0025	0.0025		0.0035	0.0005	0.0066		0.0025	0.0003	0.0049
$\delta_{11}^{\left(2\right)}$	0.8448	0.0102	0.0102		0.8628	0.8411	0.8832		0.8427	0.8174	0.8655
$\omega_{20}^{\left(1\right)}$	0.1986	0.0243	0.0243		0.0685	0.0457	0.0938		0.1229	0.0857	0.1593
$\alpha_{21}^{\left(1\right)}$	0.0886	0.0086	0.0086		0.0742	0.0578	0.0945		0.1115	0.0883	0.1343
$\beta_{21}^{\left(1\right)}$	0.0310	0.0124	0.0124		0.0127	0.0032	0.0231		0.0149	0.0031	0.0279
$\delta_{21}^{\left(1\right)}$	0.9002	0.0140	0.0140		0.9164	0.8854	0.9391		0.8462	0.8143	0.8775
$\omega_{20}^{\left(2\right)}$	0.0435	0.0146	0.0146		0.0214	0.0037	0.0417		0.0176	0.0025	0.0381
$\alpha_{21}^{\left(2\right)}$	0.0422	0.0058	0.0058		0.0539	0.0424	0.0664		0.0650	0.0549	0.0769
$\beta_{21}^{\left(2\right)}$	0.0123	0.0052	0.0052		0.0058	0.0006	0.0122		0.0061	0.0012	0.0119
$\delta_{21}^{\left(2\right)}$	0.8589	0.0133	0.0133		0.8709	0.8462	0.8923		0.8766	0.8514	0.8970
$\theta_{1}^{\left(1\right)}$	0.6106	0.0155	0.0155		0.8121	0.7590	0.8588		0.3322	0.2221	0.4398
$\theta_{2}^{\left(1\right)}$	0.0407	0.0036	0.0036		0.0636	0.0492	0.0774		0.1214	0.0944	0.1499
$\theta_{1}^{\left(2\right)}$	0.9163	0.0158	0.0158		0.9664	0.9447	0.9801		0.9279	0.8839	0.9620
$\theta_{2}^{\left(2\right)}$	0.0503	0.0046	0.0046		0.0124	0.0037	0.0221		0.0340	0.0226	0.0447

**Table 8:** Geweke diagnostic of all parameters for DS1, DS2, and DS3 based on the BHAR(1)–GJR–GARCH(1,1) model.

Parameter	DS1			DS2			DS3	
	Statistic	*p*-Value		Statistic	*p*-Value		Statistic	*p*-Value
$\phi_{10}^{\left(1\right)}$	−0.0597	0.9524		−0.8079	0.4192		−0.0636	0.9493
$\phi_{20}^{\left(1\right)}$	−0.1791	0.8579		−0.3346	0.7379		−0.0162	0.9870
$\phi_{11}^{\left(1\right)}$	1.3573	0.1747		−1.5891	0.1120		−0.2181	0.8274
$\phi_{12}^{\left(1\right)}$	0.7764	0.4375		1.7514	0.0799		−0.4235	0.6720
$\phi_{21}^{\left(1\right)}$	0.5771	0.5639		−2.2308	0.0257		−0.0380	0.9697
$\phi_{22}^{\left(1\right)}$	0.6501	0.5156		1.8316	0.0670		−0.7052	0.4807
$\phi_{10}^{\left(2\right)}$	1.8243	0.0681		0.4597	0.6457		−0.7424	0.4579
$\phi_{20}^{\left(2\right)}$	1.5591	0.1190		1.8774	0.0605		−0.6209	0.5346
$\phi_{11}^{\left(2\right)}$	0.1958	0.8448		−0.6016	0.5474		−0.9727	0.3307
$\phi_{12}^{\left(2\right)}$	−1.1547	0.2482		1.5224	0.1279		0.3103	0.7564
$\phi_{21}^{\left(2\right)}$	1.0562	0.2909		−1.2477	0.2121		−0.5624	0.5738
$\phi_{22}^{\left(2\right)}$	−1.9255	0.0542		−0.6378	0.5236		−0.0487	0.9611
$r_{L}$	−1.1629	0.2449		−2.8326	0.0046		1.8200	0.0688
$r_{U}$	−0.2210	0.8251		0.2319	0.8166		1.0950	0.2735
$\nu_{1}$	−1.1139	0.2653		−0.9501	0.3421		0.9806	0.3268
$\nu_{2}$	−1.6291	0.1033		0.0019	0.9985		0.1521	0.8791
$\rho^{\left(1\right)}$	−0.7965	0.4258		1.1421	0.2534		−1.6976	0.0896
$\rho^{\left(2\right)}$	1.2195	0.2227		−0.9170	0.3591		1.2624	0.2068
$\omega_{10}^{\left(1\right)}$	−0.9039	0.3660		−0.3661	0.7143		0.0221	0.9824
$\alpha_{11}^{\left(1\right)}$	0.9563	0.3389		−1.2333	0.2174		0.1894	0.8498
$\beta_{11}^{\left(1\right)}$	−1.3752	0.1691		1.0816	0.2794		1.2501	0.2113
$\delta_{11}^{\left(1\right)}$	−0.0239	0.9809		−0.4108	0.6813		−0.8739	0.3822
$\omega_{10}^{\left(2\right)}$	0.2948	0.7682		−0.4945	0.6209		0.4263	0.6699
$\alpha_{11}^{\left(2\right)}$	0.0896	0.9286		−2.0540	0.0400		−0.2839	0.7765
$\beta_{11}^{\left(2\right)}$	−0.0769	0.9387		0.9074	0.3642		1.8353	0.0665
$\delta_{11}^{\left(2\right)}$	−0.4204	0.6742		−0.8867	0.3753		0.3284	0.7426
$\omega_{20}^{\left(1\right)}$	1.6858	0.0918		0.6269	0.5307		−1.2515	0.2107
$\alpha_{21}^{\left(1\right)}$	−0.8687	0.3850		1.4275	0.1534		−1.1246	0.2608
$\beta_{21}^{\left(1\right)}$	1.2312	0.2182		−0.6642	0.5066		0.7641	0.4448
$\delta_{21}^{\left(1\right)}$	−1.3606	0.1736		−0.5054	0.6133		0.5095	0.6104
$\omega_{20}^{\left(2\right)}$	0.0002	0.9999		0.4037	0.6864		−0.1893	0.8499
$\alpha_{21}^{\left(2\right)}$	−1.6979	0.0895		0.6527	0.5139		−0.6733	0.5008
$\beta_{21}^{\left(2\right)}$	1.2623	0.2068		1.0262	0.3048		−0.4108	0.6812
$\delta_{21}^{\left(2\right)}$	0.0031	0.9975		−1.2335	0.2174		1.0696	0.2848
$\theta_{1}^{\left(1\right)}$	−1.0077	0.3136		0.2864	0.7746		−0.2729	0.7850
$\theta_{2}^{\left(1\right)}$	0.2581	0.7963		−0.6037	0.5460		0.1467	0.8834
$\theta_{1}^{\left(2\right)}$	0.6031	0.5465		0.4883	0.6253		−1.0627	0.2879
$\theta_{2}^{\left(2\right)}$	0.4767	0.6336		−1.0663	0.2863		1.9815	0.0475

**Table 9:** Geweke diagnostic of all parameters for DS1, DS2, and DS3 based on the BHAR(1)–QGARCH(1,1) model.

Parameter	DS1			DS2			DS3	
	Statistic	*p*-Value		Statistic	*p*-Value		Statistic	*p*-Value
$\phi_{10}^{\left(1\right)}$	−0.2382	0.8117		0.8946	0.3710		0.4246	0.6711
$\phi_{20}^{\left(1\right)}$	−0.5397	0.5894		0.4807	0.6308		0.2105	0.8332
$\phi_{11}^{\left(1\right)}$	−0.3970	0.6914		1.0016	0.3165		0.4172	0.6765
$\phi_{12}^{\left(1\right)}$	0.3031	0.7618		−0.4317	0.6659		−0.0186	0.9852
$\phi_{21}^{\left(1\right)}$	−0.9387	0.3479		0.0066	0.9947		0.6520	0.5144
$\phi_{22}^{\left(1\right)}$	0.6678	0.5043		0.3082	0.7579		−0.0690	0.9450
$\phi_{10}^{\left(2\right)}$	−0.8183	0.4132		−1.0205	0.3075		1.2391	0.2153
$\phi_{20}^{\left(2\right)}$	−1.3403	0.1802		−0.6150	0.5386		0.8368	0.4027
$\phi_{11}^{\left(2\right)}$	0.9480	0.3431		0.5433	0.5869		−0.8564	0.3918
$\phi_{12}^{\left(2\right)}$	0.3792	0.7045		0.1913	0.8483		−0.3520	0.7248
$\phi_{21}^{\left(2\right)}$	1.0261	0.3048		−0.1062	0.9154		0.2221	0.8242
$\phi_{22}^{\left(2\right)}$	−0.1441	0.8854		0.2735	0.7845		−0.6287	0.5295
$r_{L}$	0.1648	0.8691		−2.0563	0.0398		0.5816	0.5608
$r_{U}$	−0.6512	0.5149		0.4157	0.6777		−0.8724	0.3830
$\nu_{1}$	−0.0228	0.9818		−0.6572	0.5110		−0.1623	0.8711
$\nu_{2}$	0.2329	0.8159		−0.3896	0.6969		−0.7089	0.4784
$\rho^{\left(1\right)}$	−0.6398	0.5223		0.4073	0.6838		−0.3435	0.7312
$\rho^{\left(2\right)}$	0.5406	0.5888		0.3116	0.7554		0.3015	0.7630
$\omega_{10}^{\left(1\right)}$	−0.2259	0.8213		0.5642	0.5726		−1.3067	0.1913
$\alpha_{11}^{\left(1\right)}$	0.1495	0.8811		0.8659	0.3866		0.5879	0.5566
$\beta_{11}^{\left(1\right)}$	0.9298	0.3525		−0.2060	0.8368		−1.9950	0.0460
$\delta_{11}^{\left(1\right)}$	−0.1064	0.9153		−0.6188	0.5360		−0.2741	0.7840
$\omega_{10}^{\left(2\right)}$	−0.2187	0.8269		0.0908	0.9277		0.6828	0.4947
$\alpha_{11}^{\left(2\right)}$	−0.6417	0.5211		0.0376	0.9700		0.8227	0.4107
$\beta_{11}^{\left(2\right)}$	0.2499	0.8027		1.5369	0.1243		0.7921	0.4283
$\delta_{11}^{\left(2\right)}$	0.5778	0.5634		−0.9276	0.3536		−1.1088	0.2675
$\omega_{20}^{\left(1\right)}$	0.0092	0.9927		−0.4402	0.6598		0.2285	0.8193
$\alpha_{21}^{\left(1\right)}$	0.8557	0.3922		−1.3612	0.1734		0.3195	0.7494
$\beta_{21}^{\left(1\right)}$	−1.1870	0.2352		−0.9613	0.3364		0.8448	0.3982
$\delta_{21}^{\left(1\right)}$	−0.7819	0.4343		1.3219	0.1862		−0.0380	0.9697
$\omega_{20}^{\left(2\right)}$	−0.9052	0.3654		−0.1715	0.8638		1.4796	0.1390
$\alpha_{21}^{\left(2\right)}$	−0.8971	0.3697		0.0572	0.9544		0.3983	0.6904
$\beta_{21}^{\left(2\right)}$	1.0325	0.3018		−0.1454	0.8844		−0.6278	0.5301
$\delta_{21}^{\left(2\right)}$	0.7220	0.4703		0.1081	0.9139		−0.9705	0.3318
$\theta_{1}^{\left(1\right)}$	−0.1814	0.8561		0.5558	0.5783		0.3816	0.7028
$\theta_{2}^{\left(1\right)}$	2.2145	0.0268		−0.3652	0.7150		−1.0568	0.2906
$\theta_{1}^{\left(2\right)}$	−0.9347	0.3500		−0.4136	0.6792		−0.0394	0.9686
$\theta_{2}^{\left(2\right)}$	0.6555	0.5121		1.1169	0.2640		−1.2179	0.2233

**Table 10:** VaR predictions and backtesting results at the 1% level with 300 out-of-sample periods based on the BHAR(1)–GJR–GARCH(1,1), BHAR(1)–QGARCH(1,1), and BHAR(1)–GARCH(1,1) models.

	BHAR(1)–GJR–GARCH(1,1)						BHAR(1)–QGARCH(1,1)						BHAR(1)–GARCH(1,1)				
	1%			*p*-Value			1%			*p*-Value			1%			*p*-Value	
	No	VRate		UC	CC		No	VRate		UC	CC		No	VRate		UC	CC
DS1																	
S&P500	6	2.00%		0.125	0.273		8	2.67%		0.016	0.044		8	2.67%		0.016	0.045
GS	1	0.33%		0.178	0.402		1	0.33%		0.178	0.401		4	1.33%		0.016	0.045
DS2																	
S&P500	6	2.00%		0.125	0.273		8	2.67%		0.016	0.045		8	2.67%		0.016	0.044
ICE	1	0.33%		0.178	0.401		1	0.33%		0.178	0.402		4	1.33%		0.581	0.813
DS3																	
S&P500	7	2.33%		0.048	0.119		6	2.00%		0.125	0.273		9	3.00%		0.005	0.015
BAC	2	0.67%		0.537	0.815		2	0.67%		0.537	0.815		2	0.67%		0.537	0.815

**Table 11:** VaR predictions and backtesting results at the 5% level with 300 out-of-sample periods based on the BHAR(1)–GJR–GARCH(1,1), BHAR(1)–QGARCH(1,1), and BHAR(1) - GARCH(1,1) models.

	BHAR(1)–GJR–GARCH(1,1)						BHAR(1)–QGARCH(1,1)						BHAR(1)–GARCH(1,1)				
	5%			*p*-Value			5%			*p*-Value			5%			*p*-Value	
	No	VRate		UC	CC		No	VRate		UC	CC		No	VRate		UC	CC
DS1																	
S&P500	17	5.67%		0.604	0.313		17	5.67%		0.604	0.313		17	5.67%		0.604	0.873
GS	16	5.33%		0.793	0.507		14	4.67%		0.789	0.344		16	5.33%		0.793	0.507
DS2																	
S&P500	17	5.67%		0.604	0.313		17	5.67%		0.604	0.313		17	5.67%		0.604	0.873
ICE	16	5.33%		0.793	0.507		14	4.67%		0.789	0.344		16	5.33%		0.793	0.507
DS3																	
S&P500	18	6.00%		0.44	0.739		17	5.67%		0.604	0.313		16	5.33%		0.793	0.391
BAC	13	4.33%		0.588	0.478		13	4.33%		0.588	0.478		11	3.67%		0.267	0.355

**Table 12:** The backtesting measures by the authors of [26] for the estimated marginal expected shortfall based on 300 out-of-sample periods.

		DS1		DS2		DS3
At 1%						
BHAR(1)–GJR–GARCH(1,1)		**1.855**		**1.855**		2.953
BHAR(1)–QGARCH(1,1)		1.870		1.870		**2.941**
BHAR(1)–GARCH(1,1)		2.055		2.055		2.960
At 5%						
BHAR(1)–GJR–GARCH(1,1)		**1.195**		**1.195**		1.693
BHAR(1)–QGARCH(1,1)		1.253		1.253		**1.664**
BHAR(1)–GARCH(1,1)		1.401		1.401		1.830

The bold values represent the best model.

## Data Availability

The data presented in this study are available on request from the corresponding author.
